# Automated analysis of a large-scale paediatric dataset illustrates the interdependent relationship between epilepsy and sleep

**DOI:** 10.1038/s41598-023-39984-9

**Published:** 2023-08-08

**Authors:** Jelena Skorucak, Bigna K. Bölsterli, Sarah Storz, Sven Leach, Bernhard Schmitt, Georgia Ramantani, Reto Huber

**Affiliations:** 1 Child Development Center, University Children’s Hospital Zurich, University of Zurich, Zurich, Switzerland; 2 Children’s Research Center, University Children’s Hospital Zurich, University of Zurich, Zurich, Switzerland; 3 Department of Pediatric Neurology, University Children’s Hospital Zurich, University of Zurich, Zurich, Switzerland; 4 Department of Pediatric Neurology, Children’s Hospital of Eastern Switzerland, St. Gallen, Switzerland; 5 Department of Child and Adolescent Psychiatry and Psychotherapy, Psychiatric Hospital, University of Zurich, Zurich, Switzerland

**Keywords:** Epilepsy, Circadian rhythms and sleep

## Abstract

Slow waves are an electrophysiological characteristic of non-rapid eye movement sleep and a marker of the restorative function of sleep. In certain pathological conditions, such as different types of epilepsy, slow-wave sleep is affected by epileptiform discharges forming so-called “spike-waves”. Previous evidence shows that the overnight change in slope of slow waves during sleep is impaired under these conditions. However, these past studies were performed in a small number of patients, considering only short segments of the recording night. Here, we screened a clinical data set of 39′179 pediatric EEG recordings acquired in the past 25 years (1994–2019) at the University Children’s Hospital Zurich and identified 413 recordings of interest. We applied an automated approach based on machine learning to investigate the relationship between sleep and epileptic spikes in this large-scale data set. Our findings show that the overnight change in the slope of slow waves was correlated with the spike-wave index, indicating that the impairment of the net reduction in synaptic strength during sleep is spike dependent.

## Introduction

Sleep plays a vital role in many physiological functions, such as memory consolidation^1^, synaptic plasticity^2–4^, and brain metabolic waste clearance^5,6^. These functions are presumably underlying the fact that sleep is closely related to behavioral performance: insufficient sleep restoration during the night will lead to behavioral deficits during the following day^7^. Importantly, only sleep can revert these performance deficits. Non-rapid eye-movement (NREM) sleep including deep sleep is essential for the restorative functions of sleep^8–11^. Although the mechanisms underlying the restorative function of sleep are still not fully understood, according to the synaptic homeostasis hypothesis (SHY), sleep plays a role in synaptic plasticity: Synaptic strength needs to be reduced during sleep to renormalize the experience-dependent increases that occur during wakefulness^12^. Human, animal and modelling data shows that the slope of slow-waves is the most direct EEG measure for synaptic strength^13–15^. As expected based on the predictions of SHY, numerous studies have shown an overnight decrease in the slope of slow waves^15–17^, which is reflective of an overnight reduction in synaptic strength. In childhood epilepsies with a spike-wave activation during sleep, epileptic spikes invade NREM sleep. For decades it has been postulated that these spikes are the origin of cognitive and behavioral deficits these patients suffer from^18–20^. These impairments can be as dramatic as loss of language abilities or a global developmental regression^20,21^. Recent studies provide compelling evidence that the overnight decrease in slope is critically dampened in childhood epilepsies with spike-wave activation during sleep^22–25^. Interestingly, these same patient group shows significant deficits in sleep-dependent memory consolidation^26^. It is tempting to assume that impaired consolidation of newly learned information might lead with time to the patient’s behavioral and cognitive deficits. One of the key limiting factor for this research topic is the fact that so far sleep and epilepsy in pediatric patients has been investigated in only small-size datasets^22–25^. Investigating the effect of spikes on sleep in large-scale datasets is only feasible by implementing an automated approach. Furthermore, if cognitive impairment in epilepsy can be linked to both compromised overnight decrease in slow wave slope and spikes in sleep, other disorders may also benefit from this approach. This automated pipeline can be implemented to investigate sleep- and/or spike-related deficits in disorders with increased incidences for epileptic spikes, such as Alzheimer’s disease (memory deficit), or attention-deficit hyperactivity disorder (ADHD, attention deficit)^27,28^. The identification of specific sleep- and/or spike-related deficits in these disorders can help to disentangle their underlying pathomechanisms. Ultimately, an automated pipeline for sleep and spike detection may be incorporated into clinical diagnostics providing new and easily accessible information to clinicians. The implementation of this novel tool can support developing and establishing new and emerging treatment options, such as the increasingly popular acoustic stimulation during sleep that has shown promising results in patients with epilepsy^29^.

In this study, we aimed to investigate the relationship between sleep and epilepsy in a large-scale pediatric dataset acquired in the past 25 years at the University Children’s Hospital Zurich. In order to achieve that we developed a fully automated analysis pipeline for spike detection and the assessment of changes in slow wave slope as a marker for the overnight reduction in synaptic strength (SOMNIDEX; **S**ynaptic ren**O**r**M**alizatio**N I**n**DEX**).

## Methods

In a first step, the automated methods for sleep and epileptic spike detection were developed based on a small-scale dataset, carefully scored by clinical neurophysiology experts. In a next step, these automated methods were applied to a large-scale dataset, not scored by experts. This was a retrospective study, approved by Zurich Cantonal Ethics Committee BASEC-Nr. 2019–00,165. Informed consent was present for all subjects from their legal guardians. All methods were carried out in accordance with relevant Swiss guidelines and regulations.

### Small-scale dataset

A dataset pooled from several previous studies^22–24,30^ included 39 pediatric patients with epilepsies with a spike-wave activation during sleep was used for the development and testing of the algorithms for automatic sleep and spike detection. Sleep was scored by an expert in 20-s epochs during the entire recording night according to AASM criteria^31^. Spikes were marked by an expert (SS) in a 10-min EEG segment for each patient, starting after first sleep stage 2. Further, in order to estimate inter-rater variability, 1-min EEG segments were marked by 2 experts (SS and BS) in 20 recordings. Automated methods for spike and sleep detection were developed in this dataset and then applied to the large-scale dataset.

### Large-scale dataset

**Figure 1:**
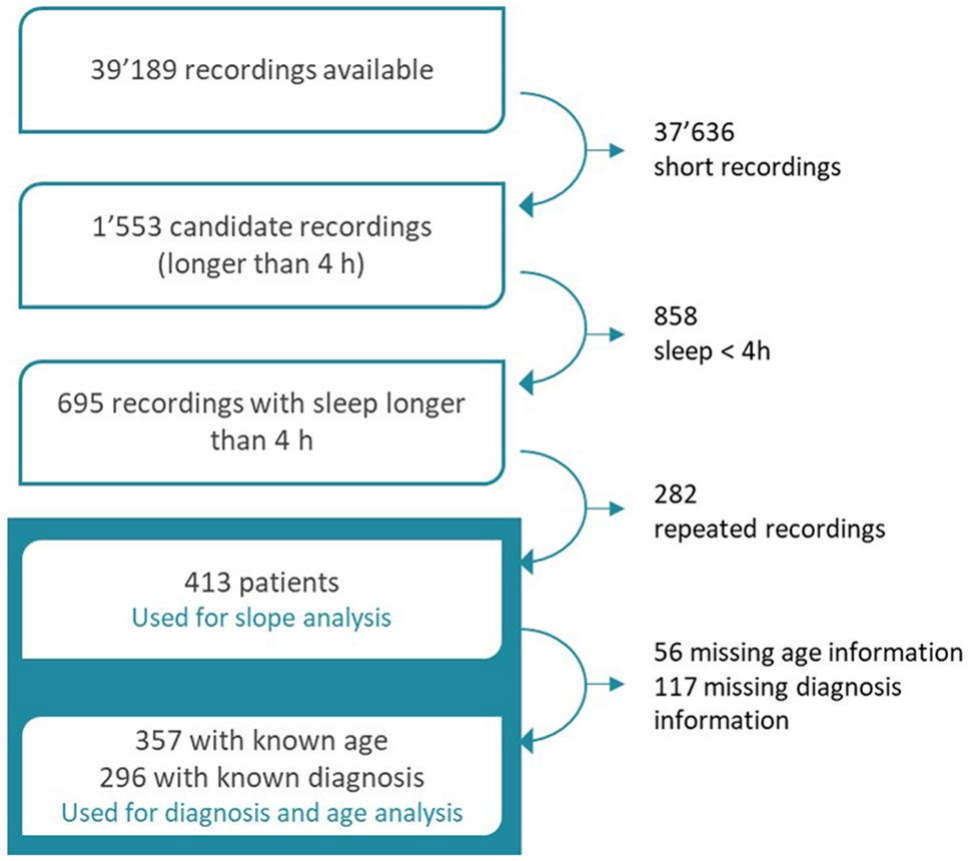
The number of recordings that were available and the number of patients that were considered for analysis.

A data set containing 39′179 EEG recordings performed at the University Children’s Hospital Zurich in 1994–2019 was available for analysis (Fig. 1, available recordings). These EEG recordings did not contain systematic annotations of sleep or spikes. In the first step, we considered recordings with duration ≥ 4 h (candidate recordings), since these were more likely to contain sleep episodes. Of 1′553 candidate recordings, 695 recordings included sleep recordings of ≥ 4 h. Other than sleep duration, no other exclusion criteria were applied. Random exclusion of repeat recordings from the same patient, resulted in 413 recordings (each from a single patient) included for slope of slow waves analysis. Exclusion of patients with age and diagnosis which were not electronically accessible in an automated fashion resulted in 357 patients included for age analysis and 296 for diagnosis analysis.

### EEG recordings and data pre-processing

EEG electrodes were placed according to the international 10–20 system. Recordings were performed at a 128 or 256 sampling rate by the Deltamed^®^ (Paris, France) EEG system. Data were exported to the European Data Format (EDF) and further processed in MATLAB R2020a (The Math Works Inc., Natick, MA). The following filters were applied: a bandpass 0.3–40 Hz, and a notch filter at 50 Hz. EEG data were re-referenced to the contralateral mastoids, and downsampled to 128 Hz, where applicable.

### Classifiers

We developed two classifiers: (1) an automatic epileptic spike detector, and (2) an automatic sleep detector. For both classifiers we applied a long short-term memory (LSTM) recurrent neural network. We used recurrent neural networks as they are taking the temporal structure into account and therefore have a good performance for time series data^32^. The small-scale dataset, used for classifier development, was divided into a training (27 patients), validation (6 patients), and testing dataset (6 patients). Models were optimized by manual tuning of (1) the number of neurons in the hidden layer, (2) the number of hidden layers, and (3) the inclusion and probability of dropout layers. The best performing model was then used for the test set applied to the large-scale dataset.

### Epileptic spike detector

The epileptic spike detector was developed based on expert scoring and raw data. The structure of our LSTM was as follows: an input layer (1 neuron), two LSTM layers (128 neurons each) each followed by a dropout layer (dropout probability 0.1), followed by a fully connected layer (2 neurons), a softmax layer, and a classification output layer. Six training epochs were applied, i.e., the entire training data were passed through the neural network six times. The adaptive moment estimation optimization algorithm (Adam) was used to update network weights during training^33^. The input of the LSTM consisted of a moving time window of 1/16 s (16 samples; step 125 ms).

### Sleep detector

Since overnight change in slope of slow waves depends on NREM sleep stages 2 and 3^8–11^, we limited our automated scoring to the detection of NREM sleep stages 2 and 3. Hence, we identified only two classes: NREM sleep stages 2 or 3, and all other stages (REM sleep, wake and NREM sleep stage 1). The classifier was developed based on the expert sleep scoring and engineered features. We used feature engineering to extract quantifiable properties of the sleep EEG, calculated from power spectral analysis of three EEG channels: F3-M2, C3-M2, and O1-M2 as: power in alpha (8–12 Hz), sigma (i.e., spindle range; 12–16 Hz), delta (i.e. slow wave activity, SWA, 0.75–4.5 Hz), and artifact range (30–40 Hz). Spectral analysis of EEG channels was performed on consecutive 20 s epochs (FFT, Tukey window [*r* = 0.5], average of five 4-s epochs; matched with sleep stages), resulting in a 0.25 Hz frequency resolution. As epileptic spikes may distort the power spectra, spectral analysis was performed after removing epileptic spikes from the data, as detected by our spike detector. The structure of the LSTM classifier was as follows: an input layer (12 neurons), two LSTM layers (64 neurons each), each followed by a dropout layer (dropout probability 0.1), followed by a fully connected layer (two neurons), a softmax layer, and a classification output layer. Twenty training epochs were applied. The input of the LSTM consisted of a moving time window of 11 sleep epochs (220 s; step: one sleep epoch or 20 s).

### Assessment of classifier performance

We assessed the performance of the classifiers by determining specificity, sensitivity, precision, accuracy, and the Cohen’s kappa coefficient^34–37^. We focused on Cohen’s kappa coefficient, as it is a robust measure, accounting for the possibility of the agreement occurring by chance^36^. We interpreted the performance results for Cohen’s kappa using Landis and Koch levels [60]: < 0.00—poor; 0.00–0.20—slight; 0.21–0.40—fair; 0.41–0.60—moderate; 0.61–0.80—substantial; 0.81–1.00—almost perfect identification^38^. Overall performance measures across all patients (pooled data) and mean values across patients were reported.

### Assessment of inter-scorer variability for spike detection

Out of 39 patient recordings, 20 were scored independently by 2 different experts. These records were randomly selected. One minute of EEG was scored for epileptic spikes. Performance measures were calculated in the same way as for the classification algorithms.

### Estimation of SOMNIDEX

SOMNIDEX was estimated by calculating overnight change in the slope of slow waves during NREM sleep. Numerous studies have shown that the slope of slow waves best reflects sleep-dependent changes in neuronal network activity, presumably underlying the reduction in synaptic strength during sleep^13–15,39^.

Slow-wave detection was performed in line with Riedner et al.^15^ First, the signal was band-pass filtered (0.5–4.0 Hz, stopband 0.1 and 10 Hz, Chebyshev Type II filter). Negative deflections between two zero-crossings were identified as slow-waves if they were separated by 0.25–1.0 s. The ascending slope of slow waves was determined by calculating the amplitude divided by the time from the most negative peak to the second zero-crossing (Figure 3A). As it is unknown if slow waves directly associated with epileptic spikes, so called spike-wave complexes, contribute to sleep homeostasis, all slow waves occurring in a window of 0.5 s after the epileptic spike were excluded from further analyses, in line with previous studies^22–24,30^. The slope of slow waves was calculated in the first hour (FH) and last hour (LH) of sleep. Instead of taking into account a fixed time period, in order to achieve a fair comparison across patients and between first and last hour of sleep, fixed amounts of NREM sleep stages 2 and 3 epochs was taken into account for this calculation, i.e. the first and last 180 20-s epochs. To account for differences in slopes due to the overnight difference in amplitude, slow waves were matched by amplitude in the first and last hour of sleep, as proposed by Jaramillo et al.^17^ In case there were less than 250 matched waves remaining after the amplitude matching procedure, this patient was excluded from the analysis (13 patients). The overnight change in the slope of slow waves reflecting SOMNIDEX was calculated as (LH-FH)/FH. Therefore, a negative overnight change indicates the expected slope decline across sleep, while no negative overnight change or a positive change indicates a pathological condition.

All analyses were performed on one EEG channel which was automatically selected as the focus of the epilepsy. The focus channel was determined as the channel with the highest spike wave index (SWI; number of spikes per 10-s time interval).

Variables are described as mean and standard deviation of the mean, and median with interquartile range.

### Correlation analysis

Correlations were performed using Spearman’s correlation coefficient.

## Results

We created an automated pipeline for data processing, where all calculations, including pre-processing, spike detection, sleep detection, and SOMNIDEX calculation, were performed in a computerized manner, without any user dependencies.

### Spike and sleep classifiers performance

**Figure 2:**
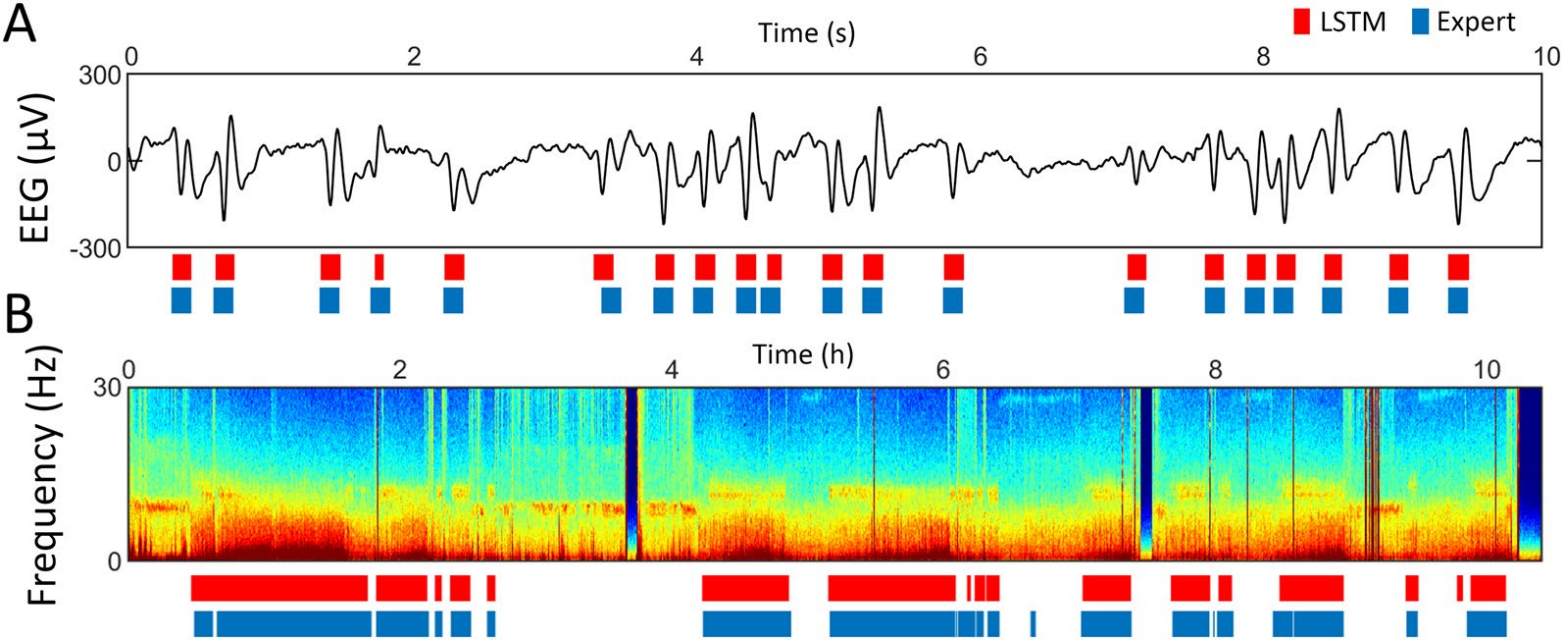
Spike detection (top, ten-second EEG trace with epileptic activity) and sleep detection (bottom, spectrogram of a 10-h night EEG recording with frequency at the y-axis and time at x-axis, and warmer colors representing higher power values) of a patient with spike-wave epilepsy. Expert scoring is depicted in blue and automatic detection (LSTM) in red, below the respective graph.

Two classifiers were developed: an automatic detector of epileptic spikes, and an automatic detector of sleep (NREM sleep stages 2 and 3). As exemplified in the EEG trace including epileptic activity (Fig. 2A) there was a good overlap between expert and automated spike detection. The time–frequency plot of an exemplary night (Fig. 2B) shows a similarly good overlap between the expert and automated NREM sleep stages 2 and 3 classification. When quantifying these overlaps (Supplementary Table), both the automatic detection of epileptic spikes and the automatic detection of sleep showed good performance, with a Cohen’s kappa coefficient of 0.72 and 0.80, and an accuracy of 92.3% and 90.3% respectively. On a validation dataset, Cohen’s kappa coefficient was 0.73 for spike detection and 0.71 for sleep detection, while accuracy was 93.7% and 86.4%. Cohen’s kappa of inter-scorer reliability calculated in 1-min EEG segments of 20 patients was 0.71 for the spike detection, indicating similar agreement between two experts as between one expert and the algorithm.

### Slope of slow waves decreases across age in children

**Figure 3:**
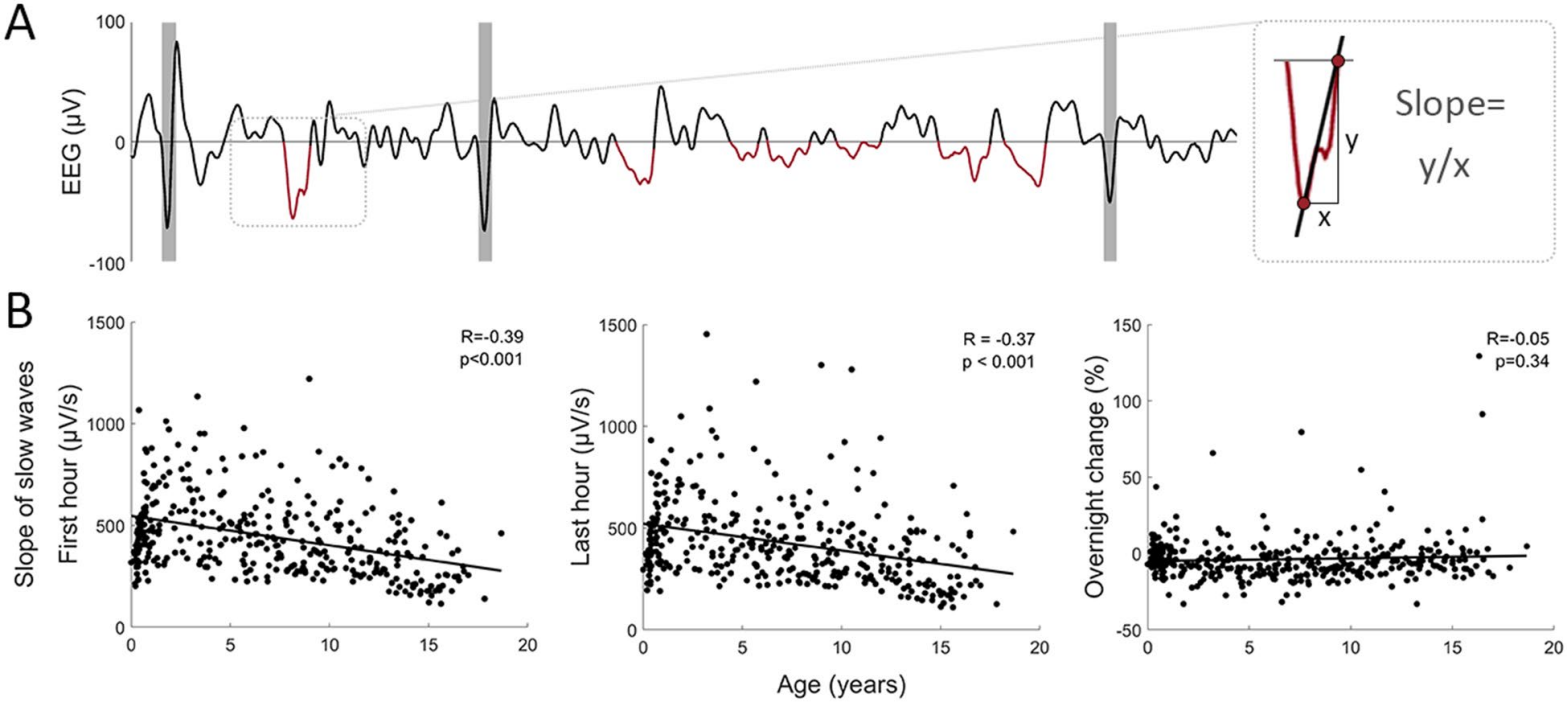
Slope of slow waves. (**A**) 10-s EEG trace with epileptic spikes in shaded gray, and slow waves in dark red, as identified by our automated methods. Note that slow waves occurring 0.5 s after the spike were not considered for analysis. Also, only slow waves that were matched in the amplitude matching procedure between the first and last hour of NREM sleep were further analyzed. The slope was calculated as the ascending slope of slow waves from the negative peak to the zero crossing. (**B**) Correlation between age and slope of slow waves in the first hour (FH; R =  − 0.39, *p* < 0.001), in the last hour of sleep (LH; R =  − 0.37, *p* < 0.001), and overnight change in slope (not significant). N = 357.

The slope of slow waves was calculated for the first and last hour of NREM sleep, as illustrated in Fig. 3. As an indirect validation of our methods, we investigated whether we could replicate previous findings in healthy children^17^. We assessed the correlation between the age of our patients (6.94 ± 5.11; 6.61[1.81–11.13] years) and the slow wave slopes in the first (446.08 ± 193.11; 414.78[301.53–546.54] µV/s), the last hour of NREM sleep (429.61 ± 203.46; 393.62[281.69–518.80] µV/s), and the overnight change in slope (− 3.72 ± 14.95; − 5.84[− 10.88–1.07] %). The slopes both in the first and last hour of sleep were negatively correlated with age (R =  − 0.39, *p* = 3.3 × 10^−14^ and R =  − 0.37, *p* = 4.5 × 10^−13^ respectively), and the overnight change in slope did not show a correlation with age (R =  − 0.05, *p* = 0.34).

### SOMNIDEX is impaired in children with epilepsy

**Figure 4:**
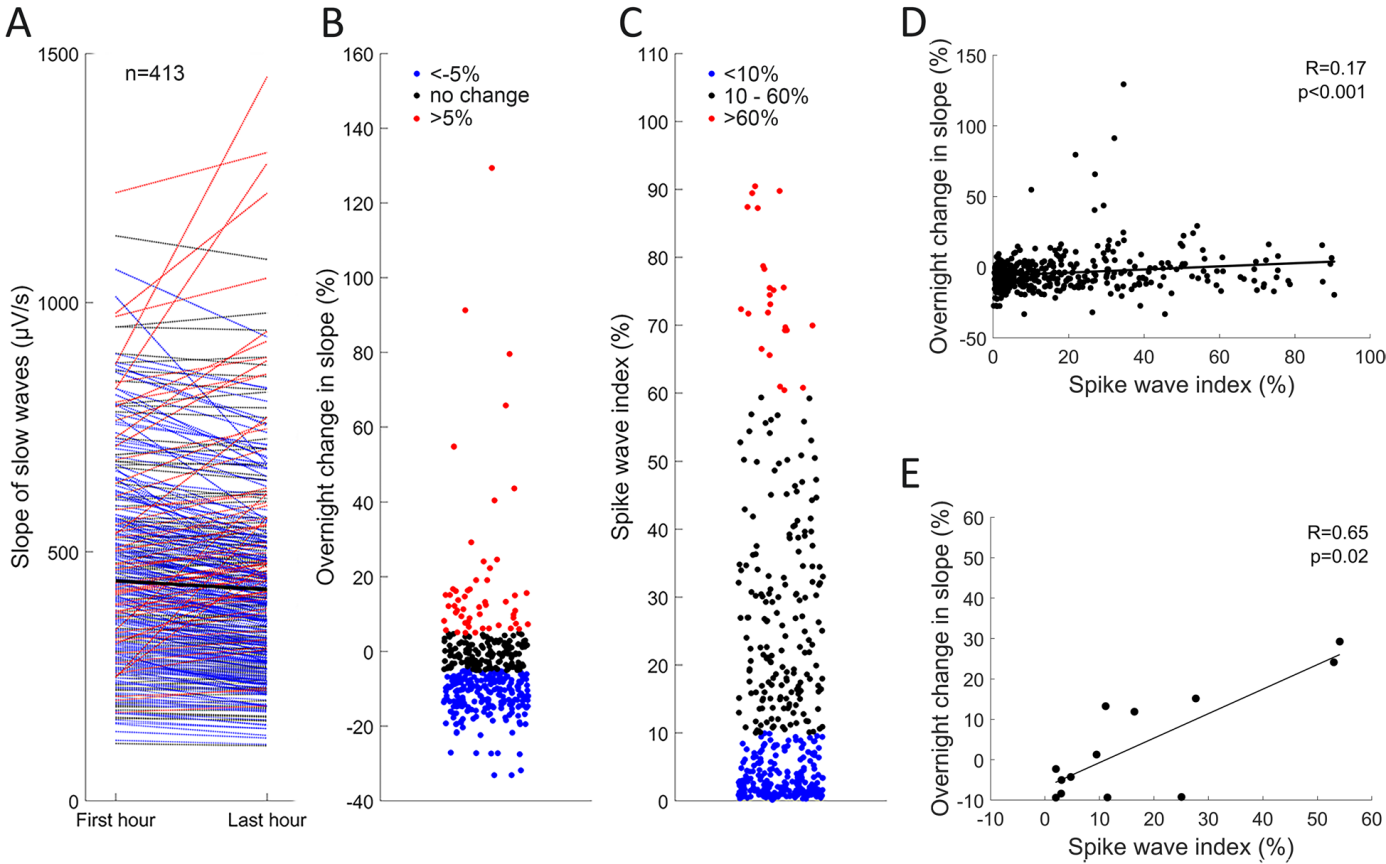
(**A**) Slope of slow waves in the first and last hour of NREM sleep (N = 413). Black line represents the average value. There was no significant difference between the slope in the first and in the last hour of sleep (two-sided Wilcoxon rank sum test, *p* > 0.05). (**B**) Overnight change in slope (higher values represent higher impairment in synaptic renormalization during sleep, i.e., positive values represent overnight increase in slope). (**C**) Spike wave index, a clinical marker of severity of epilepsy. (**D**) Correlation between the overnight change in slope and spike wave index (R = 0.17, *p* < 0.001). (**E**) Correlation between overnight change in slope and spike wave index for Lennox Gastaut patients (N = 13).

Finally, we investigated sleep aspects in our patient cohort (Fig. 4) by assessing SOMNIDEX: changes in the slope of slow waves from the first (mean ± standard deviation: 441.96 ± 190.92 µV/s; median and interquartile range: 413.01 [297.85–538.01] µV/s) to the last hour of NREM sleep (424.29 ± 199.53; 391.87 [281.47–510.38] µV/s). In the healthy population a decline in slope from the first to the last hour is expected^17,22^. We found no significant change of the slope of slow waves (− 4.04 ± 14.16; − 5.86 [− 11.29–0.78]%) in our cohort (Wilcoxon rank sum test, U = 176,791, z = 1.75, *p* = 0.08, CI = [− 0.06, 0.22], Cohen’s d = 0.09), indicating impaired synaptic renormalization during sleep. Specifically, 54% of children showed the expected overnight decrease in the slope of slow waves, but one-third (33%) of children showed no change, and 13% of children showed an overnight increase (Figs. 4A and 4B). The patients showing an overnight increase in the slope may represent a clinically relevant population, as their overnight change in slope is severely impaired.

Further, we calculated the spike wave index (SWI; 17.60 ± 20.34; 9.43[2.26–26.82]%), a clinically relevant marker of the severity of epilepsy (Fig. 4C). Similar to our observation regarding overnight slope changes, 52% of children showed low rates of spike activity, while 42% showed high, and 6% very high rates of spike activity. Interestingly, the change in the slope of slow waves was positively correlated with the SWI (R = 0.17, *p* < 0.001), i.e., the more spikes, the stronger the impairment in synaptic renormalization during sleep. Finally, we identified Lennox Gastaut patients as the patient group showing the highest correlation between these two parameters (R = 0.65, *p* = 0.018).

## Discussion

Both classifiers showed good performance, indicating that reliable computerized detection of epileptic spikes and sleep is feasible. Similar performance in the validation and testing datasets indicate that there was no overfitting of the training model. Importantly, the spike detection model achieved the same performance as the inter-rater variability, indicating that our spike detector achieved expert level of performance. Inter-scorer agreement for sleep scoring has been previously reported in the literature, and it was not assessed in the scope of the current study. Our classification performance is in the range of previously reported classifiers in healthy volunteers and patients^40,41^, and in line with the observed inter-rater agreement for the AASM standard (0.76 as overall Cohen's kappa coefficient)^42,43^.

The entire data analysis pipeline in this study was performed in an automated manner. We analyzed approximately 50 years of EEG data (1553 candidate recordings, 19 EEG derivations), which would take approximately 90 years for an expert to annotate. To the best of our knowledge, this is the first study to investigate the relationship between sleep and epilepsy with automated methods in such a large-scale pediatric dataset. The fully automated approach has the obvious advantage of not requiring intermediate checking steps, manual tuning of parameters, or similar. This automation facilitates fast and efficient data analysis and enables the otherwise unfeasible processing of large-scale datasets. Moreover, as spike detection algorithm derives spike counts for each individual EEG channel, this approach may also permit the topographical analysis of epileptic spikes, i.e., the comparison of the SWI within various epileptic foci against the rest of the brain. This topographical mapping may also allow for an automated detection of the epileptic focus. Further, this automatic approach together with our SOMNIDEX findings are of importance for the clinical diagnostics as the entire night recording can be considered, and there may be some variability across a sleep episode. Finally, our approach allows an assessment of sleep aspects, which are currently not considered in clinical practice, and may provide important additional information about the pathophysiology of the disorder. Of course, the applicability to medical diagnostic still needs to be proven with the potential benefit of faster and more standardized diagnostics. Furthermore, automated methods in conjunction with mobile EEG devices open doors for hospital-at-home applications.

We performed several analyses to support the validity and significance of our approach:

First, we found a negative correlation between slow wave slopes and age, in line with previous studies in healthy children and adults aged 8–26 years^17^, but found no correlation between age and overnight change in slow wave slope, in contrast to these studies^17,44^. The lack of correlation between overnight changes in slow wave slope and age in our study may be attributed to the impaired overnight change in slope we observed in our cohort, since higher variability in impairment may have masked the effect of age in our dataset. Several hypotheses about the function of sleep associate sleep restoration to processes critical for learning and memory, i.e., memory consolidation^1^, synaptic plasticity^2–4^, and the brain metabolic waste clearance^5,6^. In the scope of this paper, an overnight change in the slope of slow waves was calculated. The slope of slow waves is hypothesized to represent an electrophysiological marker of synaptic strength, and an overnight decline in slope indicates synaptic down-selection, a critical process for sleep dependent learning and memory consolidation processes^45,46^. Hence, impaired overnight change in slope may reflect these fundamental processes of sleep restoration. It is thus no surprise that patients suffering from severe forms of sleep-associated spike wave epilepsies show impaired memory consolidation^30,44^. Moreover, it is speculated that the cognitive deficits of these patients may be a consequence of impaired sleep functions^20^. When we applied arbitrary cutoffs to SOMNIDEX, the overnight change in the slope of slow waves, 13% of patients had severely impaired overnight change in slope, 33% of patients showed moderately impaired overnight change in slope, and 54% of patients had normal overnight change in slope. This variability in our patient group is in line with a previous study in a small-scale dataset (N = 9)^22^ where an increased overnight slope change (severe impairment) was observed in two patients, a reduced overnight slope decline (moderate impairment) was observed in four, and an overnight slope decrease in three patients. In contrast, healthy children exhibit on average a 15% overnight decrease in the slope of slow waves^17^. Although healthy populations show some variability in the overnight change in slope, none of the included study participants showed an overnight increase in the slope of slow waves^17,22^. Therefore, we can consider an overnight increase in slope pathological, and we propose this sleep restoration marker as an additional parameter in clinical diagnostics.

Second, we analyzed an important clinical parameter—the SWI. When applying arbitrary cutoffs to our clinical marker, 6% of patients had a very high SWI (> 60%), 43% showed a SWI over 10% (but under 60%), and half (52%) of the patients had a low SWI (< 10%). The variability in SWI is in line with previous studies^30^ observing SWI in the range of 9 to 98% in 14 patients with self-limited focal epilepsies of childhood^45^. In a retrospective review^47^ of 102 children with continuous spike waves during sleep, those with SWI > 50% were more likely to present with global developmental problems, while those with SWI < 50% were more likely to manifest specific forms of neurological impairment. However^46^, the latest position paper of the International league against epilepsy (ILAE) Task Force on Nosology and Definitions^48^ proposes the term developmental and epileptic encephalopathy with continuous spike waves during sleep without any specification regarding a minimum SWI but rather the causal relation between epileptic activity and developmental regression. Nevertheless, the SWI is an objective marker for the risk to develop cognitive deficits, and we propose that the SOMNIDEX linked to the restorative function of sleep might be an additional marker more tightly linked to the pathomechanisms behind these cognitive impairments.

Third, we assessed the relationship between net reduction in synaptic strength during sleep and SWI. In line with previous results^24^ we observed a correlation between these two markers: The higher the SWI, the higher the impairment in the SOMNIDEX during sleep. This correlation was highest for Lennox Gastaut patients. Although no studies have investigated the association between SWI and overnight slope of slow waves in Lennox Gastaut patients, it should be noted that this patient group is characterized by a strong increase of spike waves during sleep, multiple seizure types, and cognitive impairment^49^. In the scope of the current study, SOMNIDEX was correlated to SWI only, however, it would be important to test the relevance of SOMNIDEX by correlating it to other clinical markers in future studies.

In conclusion, investigating sleep restoration parameters in clinical populations may provide new information about the underlying mechanisms of the disease. In this study, our methodology has been applied to patients with childhood epilepsy, but the proposed methods are applicable to any disorder related to sleep or spike alterations (e.g., Alzheimer’s, Parkinson’s, autism, ADHD). A better understanding of the underlying pathology has become increasingly important in recent years, due to the rise of new technologies enabling us to manipulate sleep, such as acoustic stimulation^50^, which may lead to new treatment options for these disorders.

### Supplementary Information

Supplementary Information.

## Data Availability

Patient data used in the scope of this study cannot be made publicly available to protect patients ‘ privacy. Data can be accessed by investigators upon reasonable request and proof of adequate ethical approval to Prof. Reto Huber.

## Code availability

The code can be accessed by investigators upon a reasonable request to the authors.
