# Supplementary material for: Automated analysis of a large-scale paediatric dataset illustrates the interdependent relationship between epilepsy and sleep
Source: Sci Rep. 2023 Aug 8;13:12882. doi: 10.1038/s41598-023-39984-9 (PMC10409812; doi:10.1038/s41598-023-39984-9)
Supplement: Supplementary file 1 — Supplementary Information. [file 41598_2023_39984_MOESM1_ESM.docx]

|  | Sensitivity | Specificity | Precision | Accuracy | Cohen’s kappa |
| --- | --- | --- | --- | --- | --- |
| Spike detector (validation) | 67.31 | 98.48 | 88.98 | 93.66 | 0.73 |
|  | 66.09 ± 6.9 | 98.48 ± 0.5 | 86.94 ± 3.8 | 93.61 ± 1.3 | 0.71 ± 0.06 |
| Spike detector (testing) | 70.41 | 97.15 | 84.57 | 92.29 | 0.72 |
|  | 69.53 ± 3.9 | 96.68 ± 1.3 | 85.05 ± 2.5 | 91.98 ± 2.1 | 0.71 ± 0.04 |
| Sleep detector (validation) | 87.30 | 84.85 | 90.23 | 86.36 | 0.71 |
|  | 87.94 ± 4.8 | 85.99 ± 4.9 | 91.18 ± 2.9 | 86.11 ± 2.9 | 0.71 ± 0.06 |
| Sleep detector (testing) | 95.23 | 83.57 | 88.65 | 90.26 | 0.80 |
|  | 95.23 ± 1.7 | 71.66 ± 13.9 | 89.40 ± 4.1 | 89.30 ± 3.7 | 0.69 ± 0.12 |

***Supplementary Table:*** *Performance of spike and sleep detection classifiers for validation and test dataset: sensitivity, specificity, precision, accuracy, and Cohen’s kappa coefficient. The classification algorithms were validated in six patients and tested in six patients. Overall performance measures across all patients (numbers without standard errors of the mean; data of all patients were pooled) and mean across patients and standard error of the mean. The performance was calculated based on the 125 ms resolution for spike detector, and one sleep epoch (20 s) for sleep detector.*
